# Centrifugation-assisted Assembly of Colloidal Silica into Crack-Free and Transferrable Films with Tunable Crystalline Structures

**DOI:** 10.1038/srep12100

**Published:** 2015-07-10

**Authors:** Wen Fan, Min Chen, Shu Yang, Limin Wu

**Affiliations:** 1 Department of Materials Science and State Key Laboratory of Molecular Engineering of Polymers, Advanced Coatings Research Center of Ministry of Education of China, Fudan University, Shanghai 200433, China; 2 Department of Materials Science and Engineering, University of Pennsylvania, 3231 Walnut Street, Philadelphia, PA 19104, USA

## Abstract

Self-assembly of colloidal particles into colloidal films has many actual and potential applications. While various strategies have been developed to direct the assembly of colloidal particles, fabrication of crack-free and transferrable colloidal film with controllable crystal structures still remains a major challenge. Here we show a centrifugation-assisted assembly of colloidal silica spheres into free-standing colloidal film by using the liquid/liquid interfaces of three immiscible phases. Through independent control of centrifugal force and interparticle electrostatic repulsion, polycrystalline, single-crystalline and quasi-amorphous structures can be readily obtained. More importantly, by dehydration of silica particles during centrifugation, the spontaneous formation of capillary water bridges between particles enables the binding and pre-shrinkage of the assembled array at the fluid interface. Thus the assembled colloidal films are not only crack-free, but also robust and flexible enough to be easily transferred on various planar and curved substrates.

Bottom-up assembly of colloidal particles has attracted considerable attention in the past decades because it allows for cost-effective and flexible fabrication of monolayer or multilayer colloidal films with predetermined microstructures, which have a wide range of actual and potential applications, including photonic crystals^1^,^2^, photonic glasses^3^,^4^,^5^, electronic devices^6^,^7^, responsive sensors^8^, macroporous materials^9^ and surface coatings with structural color or specific wettability^10^,^11^,^12^.

Self-assembly on a solid substrate is the most common strategy to fabricate colloidal films. While the solid substrate provides a support to colloidal crystallization, it may also hinder the assembly into the most energetically favorable position. For example, in gravity or centrifugal sedimentation method^13^,^14^,^15^,^16^, the rearrangement of packed particles appears to be severely restricted by their low mobility on the bottom substrate when the stabilizing factor (*e.g.* gravity or centrifugal force) exceeds the destabilizing factor (*e.g.* Brownian motion or interparticle electrostatic repulsion). Thus, the products are considerably less ordered, unless a slow attainment of equilibrium (typically hours to days) is achieved between two competing factors, causing poor assembly efficiency. Alternatively, the vertical deposition by lateral capillary attraction during solvent evaporation is effective in inducing oriented crystallization^17^. However, cracks often occur upon drying, due to the transverse tensile stress arising from the capillary-force-induced shrinkage of the pre-assembled array against a rigid substrate^18^. To avoid undesirable cracks, sol-gel precursors and monomers have been introduced to reduce the capillary effect^18^,^19^. Nevertheless, this approach is limited to some specific material systems. Spin-coating offers another method to produce crack-free colloidal films, but also requires use of refractive-index matching monomers and produces non-close-packed structures^20^. Additionally, because the hydrophilic/hydrophobic nature and surface roughness of a substrate are critical to the success of crystal growth^17^,^20^, a limited choice of substrates (*e.g.* silicone wafer or glass) are often used to deposit colloids. However, once the crystal film is formed on such substrates, it is not easy to transfer the film to another substrate (*e.g.* III-V device, lens or polymer substrate) for device integration or flexible applications.

Recently, self-assembly at liquid/fluid interface has been reported as a facile and universal strategy for constructing low-dimensional nanostructures^6^,^7^,^21^. Minimization of interfacial energy, either by spontaneous adsorption of partially hydrophobic particles^22^, or by ethanol-induced adsorption of hydrophilic particles^23^,^24^, provides the driving force to trap colloidal particles at an air/water or oil/water interface to create a monolayer film. The main advantage of interfacial assembly lies in its low resistance to lateral motion and transferability of the obtained film. However, the adsorption and binding of particles at an interface is always accompanied by the shielding of interparticle electrostatic repulsion, which is mainly responsible for long-range ordering^25^. Thus, there approaches usually have poor control over the monolayer structure and are not suitable to prepare multilayer colloidal film, since a multiple deposition process will further degrade the film quality.

In this study, we report a novel and simple centrifugation-assisted assembly method to create free-standing multilayer film from colloidal silica spheres at the liquid/liquid interfaces of three immiscible liquid phases. Our approach has several advantages: i) The present method can facilitate the binding and pre-shrinkage of the assembled array at the fluid substrate by utilizing the spontaneous formation of capillary water bridges between silica particles. This not only surmounts the cracking problem during drying of the film, but also produces a reasonably compact and robust, free-standing film, which can be easily transferred to any planar and curved substrates; ii) Centrifugal field can relax the constraint on particle hydrophobicity required for adsorption of colloidal particles to an interface without affecting the properties of interface and particles; iii) The low resistance fluid interface provides little barrier to the final equilibrium between the interfacial adsorbed particles, thus various crystal morphologies, including polycrystalline, single-crystalline and quasi-amorphous structures, can be readily obtained by independently adjusting the centrifugal force and interparticle electrostatic repulsion.

## Results and Discussion

### Fabrication of free-standing colloidal silica films

**Figure 1:**
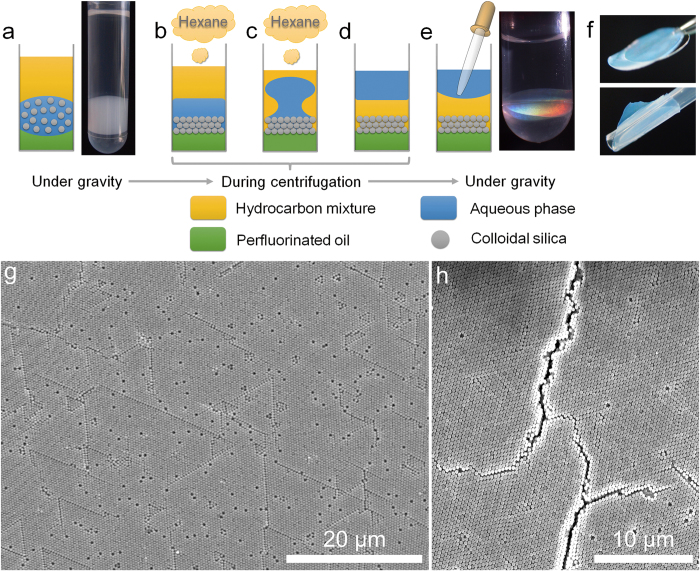
Illustration of centrifugation-assisted assembly and binding of colloidal silica at liquid/liquid interfaces. (**a**) The general state of three immiscible liquid phases under normal gravity. (**b**) During centrifugation, the perfluorinated oil/water interface is flattened, thus favoring the assembly of colloidal silica at the interface. (**c**) The aqueous phase is forced to separate from the assembled colloidal film as the hydrocarbon mixture becomes denser than the aqueous phase due to volatilization of hexane. (**d**) After dehydration of colloids, the capillary water bridges between contacting particles may lead to a binding state. (**e,f**) After removal of the aqueous phase, the free-standing colloidal film can be readily transferred to a desired substrate, even with a curved surface. Typical surface SEM images of the colloidal films prepared from 0.3 wt % aqueous suspension (**g**) by the present method after centrifugation at 18,400 g for 10 min, and (**h**) by traditional vertical deposition at 65 °C for 6 h. The silica particles have a diameter of 400 nm.

Figure 1 illustrates the centrifugation-assisted assembly and binding of monodisperse colloidal silica spheres at liquid/liquid interfaces. Briefly, perfluorinated oil (perfluorotripropylamine, 1,820 kg/m^3^), aqueous suspension of colloidal silica (~1,000 kg/m^3^), together with a mixture of hydrocarbon (~890 kg/m^3^) consisting of hexane (660 kg/m^3^), butyl acetate (880 kg/m^3^) and tetrachloroethylene (1,620 kg/m^3^) with a volume ratio of 3:3:1, were placed in a polypropylene centrifuge tube. The tube without cap was left open to the air. The three liquid phases were immiscible with each other and separated by gravity because of their different densities. Under gravity, the perfluorinated oil/water interface is slightly concave because the low surface energy perfluorinated oil preferentially wets the inner wall of the polypropylene centrifuge tube (Fig. 1a). When centrifugal acceleration of >1,000 g was exerted to the tube, the perfluorinated oil/water interface was flattened (see Fig. S1 in Supporting Information) and the colloidal silica were assembled at this interface (Fig. 1b). Meanwhile, hexane has lower boiling point (68.5 °C) than those of butyl acetate (126.1 °C) and tetrachloroethylene (121.1 °C), and would preferentially volatilize from the hydrocarbon mixture during centrifugation with the help of an air flow generated by the rotating rotor. Accordingly, the density of the hydrocarbon mixture gradually increased with the volatilization of hexane, and eventually became slightly larger than that of the aqueous phase within typically 10 min. Then, the hydrocarbon mixture sank to the interface of perfluorinated oil and aqueous phase along the inner wall of the centrifuge tube (Fig. 1c), and the assembled film was well-retained at the perfluorinated oil/hydrocarbon mixture interface by the strong centrifugal force (Fig. 1d). The resulting displacement between aqueous phase and hydrocarbon mixture not only prevented further volatilization of hydrocarbon mixture, but caused dehydration of colloidal film because the aqueous phase has a strong tendency to separate from the hydrophilic particles under a centrifugal field. Moreover, the interface of perfluorinated oil/hydrocarbon mixture was rather flat even under normal gravity due to its low interfacial tension, unlike the perfluorinated oil/water interface which returned to a slightly concave shape once the centrifugal field was removed. This shape of the configurally assembled colloidal film could be preserved after removal of the centrifugal field (Fig. 1e). More importantly, the dehydrated film was freestanding, robust and flexible enough to be transferred to both planar and curved substrates (Fig. 1f), and the colloidal film was crack-free compared to those obtained from vertical deposition, as shown in Fig. 1g,h. This is attributed to the capillary-force-induced pre-shrinkage of the assembled film at the fluid substrate, and will be discussed later.

### Control of the crystal structures

**Figure 2:**
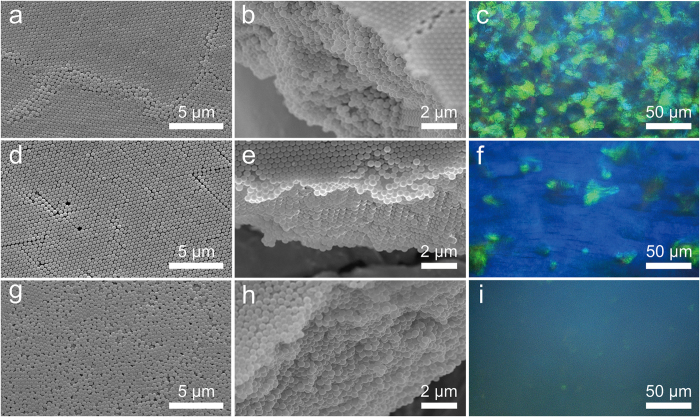
Control of the crystal morphology of the colloidal films. SEM images of the bottom surfaces and the cross-sections, and the corresponding optical microscope images of the (**a,b,c**) polycrystalline, (**d,e,f**) single-crystalline and (**g,h,i**) quasi-amorphous colloidal films assembled from 0.3 wt% of 400 nm silica sphere.

Figure 2 presents SEM images of the obtained films with polycrystalline, nearly single-crystalline and quasi-amorphous structures by tuning the surface charge (see Table S1) of silica colloids (400 nm in diameter) and the acceleration rate of centrifuge rotor. The refractive index matching between silica particles and void-filling fluid enables direct observation of the internal crystal structures of the films under a microscope. When the original colloidal suspension was rinsed with deionized water to remove excessive electrolyte and allowed a rapid acceleration to 18,400 g in 10 sec, a polycrystalline structure with obvious grain boundaries was obtained (Fig. 2a,b), displaying bright but patchy-colors which indicate different grain orientations (Fig. 2c). By increasing the acceleration time to 30 sec, the freshly washed colloids assembled into a nearly single-crystalline film (Fig. 2d,e), and the bright blue color indicates significantly increased size of the preferentially oriented grains (Fig. 2f). These so-called structural colors are attributed to constructive interference of the scattered light from periodic distribution of refractive index changes in the crystal structures, and are usually angle-dependent, creating shimmering iridescence as seen in beetle scales and birds’ feathers^1^,^2^.

When the colloids were dispersed in 5 mM NaCl solution and allowed for a rapid acceleration to 18,400 g in 10 sec, the suspension assembled into a quasi-amorphous structure with short-range ordering (Fig. 2g,h), and the bright crystalline regions were hardly observed (Fig. 2i). In contrast to the polycrystalline and single-crystalline films, the quasi-amorphous film showed uniform and angle-independent color like the skins of odonate and mandrill^26^, possibly due to strong incoherent scattering from the randomly distributed particles. This characteristic could have important applications in disordered photonics, such as random lasing and disorder-induced light localization^4^,^5^.

### Assembly mechanism

**Figure 3:**
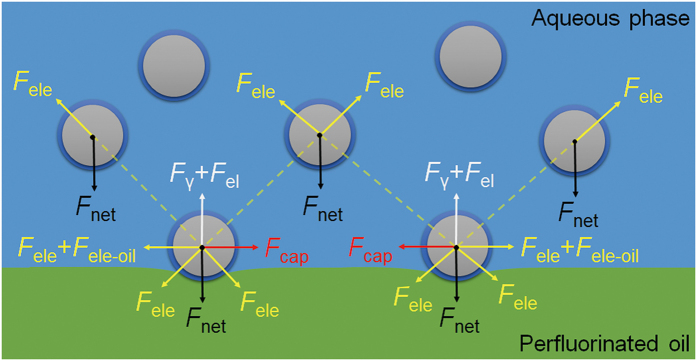
Modeling of assembly forces. Various forces that may affect the assembly of colloidal silica at the perfluorinated oil/water interface during centrifugation.

To understand the assembly mechanism of colloidal silica into different crystal structures, we investigated the forces that may contribute to the assembly process at perfluorinated oil/water interface, as shown in Fig. 3. *F*_net_ is the net centrifugal force (the centrifugal force minus the buoyancy force) acting vertically downward on each particle. *F*_γ_ is the vertical component of the interfacial tension force, which arises from the deformation of the interface around adsorbed particles, and attempts to balance the net downward force acting on the adsorbed particles and holds the particles at the interface^27^. *F*_cap_ is the centrifugation-induced capillary attraction force between two adsorbed particles, due to the overlap of the menisci formed around the particles^28^. *F*_el_ is the electrodipping force that tends to push the adsorbed particles into the phase with higher dielectric constant (water) due to the image-charge effect^25^,^29^. *F*_ele_ is the screened Coulomb repulsion between two particles arising from the overlap of electrical double layers in the aqueous phase^30^. *F*_ele-oil_ is the direct Coulomb repulsion between two adsorbed particles mediated through the nonpolar perfluorinated oil phase, owing to residual surface charges at the particle/oil interface^31^,^32^. Dipolar repulsions can also occur between two adsorbed particles through the oil phase due to the charges at the particle/water interface or the dipoles at the particle/oil interface, respectively^33^,^34^. However, it has been demonstrated theoretically and experimentally that the total repulsion between adsorbed hydrophilic particles is mainly controlled by the *F*_ele_, especially when the particle size is comparable to the Debye screening length *κ*^-1^ 31,32,33,34. Moreover, it is known that the hydration repulsion, which presumably arises from the overlap of the hydrogen-bonded hydration layers surrounding silica particles, will dominate the interaction between silica particles when surface separation is smaller than ~8.8 nm^35^. Beyond this distance, the van der Waals attraction between two adjacent particles is negligible, and the *F*_ele_ between any two particles (partially or completely immersed in the aqueous phase) was estimated to be comparable to the *F*_net_ acting on a settling particle (see Fig. S2). For simplification, the hydrodynamic drag force exerted on the adsorbed particles is ignored here.

It is clear that the centrifugal field should accelerate the deposition of colloidal particles at a heavy oil/water interface^36^. But it remains unclear whether the centrifugal field can effectively induce interfacial deformation around colloidal particles, thereby producing sufficient lateral capillary attraction to direct the assembly of adsorbed colloidal particles. Here, we first demonstrate by calculation that for small colloidal silica (*e.g.* radius ≤200 nm as considered here), the centrifugation-induced capillary attraction has little influence on the assembly process (see Figs S3 and S4).

**Figure 4:**
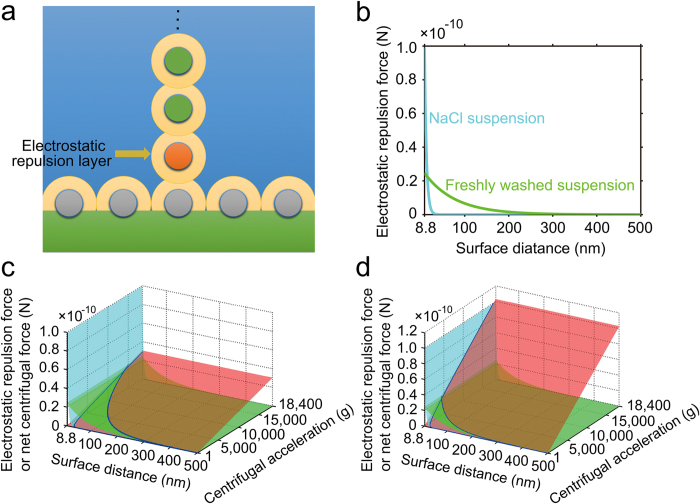
Competition interaction between centrifugal force and interparticle electrostatic repulsion. (**a**) A settling particle (orange) moving towards an adsorbed particle (gray) under the net centrifugal force acting on itself and the additional net centrifugal force from particles in the upper layer (green). (**b**) Comparison of the electrostatic repulsion force between two silica particles dispersed in 5 mM NaCl aq. solution (NaCl suspension, blue line) or deionized water (freshly washed suspension, green line). (**c,d**) Comparison of the total net centrifugal force (red surface) acting on a settling particle and the electrostatic repulsion force between two particles for NaCl-treated (blue surface) and freshly washed (green surface) suspensions, with (**c**) 4 layers and (**d**) 16 layers of upper particles acting on the settling particle, respectively.

To quantitatively understand the interaction between centrifugal force and interparticle electrostatic repulsion, we consider a settling particle moving towards an adsorbed particle under the net centrifugal force acting on itself and the additional net centrifugal force exerted by the upper particles. Figure 4 compares the total net centrifugal force acting on the settling particle and the electrostatic repulsion between two particles for NaCl-treated and freshly washed suspensions, respectively. In the early stage of centrifugation (Fig. 4a), the settling particles are increasingly concentrated towards the oil/water interface, a spontaneous disorder-to-order transition, or so-called soft Kirkwood-Alder transition^37^, would occur preferentially between the adsorbed particles once the concentration of adsorbed particles exceeds a critical value, above which the adsorbed particles would electrostatically repel each other at their equilibrium positions. Because the critical transition concentration is inversely proportional to the effective range of the repulsion^38^, we speculate that in the freshly washed suspension, as shown in Fig. 4b, the transition should begin at a relatively low concentration of adsorbed particles or at a large distance owing to the long-range electrostatic repulsion. In the NaCl-treated suspension, because the electrostatic repulsion is sufficiently screened at large distances, the transition will not be significant until the adsorbed particles closely approach one another in a random distribution.

In the freshly washed suspension, the total net centrifugal force acting on the settling particle can be strong enough to overcome the electrostatic repulsion exerted by the adsorbed particle when a few layers of upper particles (*e.g.* 4 layers) are acting on the settling particle (Fig. 4c). Thus, the settling particles are readily forced into the remaining voids between adsorbed particles until in contact with hydration layers. At this stage, the equilibrium between adsorbed particles is constantly disturbed by the impact of newly trapped particles, which grows with increasing particle concentration, in order to accommodate each other. Meanwhile, the tendency towards equilibrium can also be enhanced due to the increased electrostatic repulsion at shorter separation distances. Thus, the final structure of the adsorbed layer should be a balance of competition between these two opposing processes, namely disordering and crystallization. In the case of rapid deposition, the freshly washed particles should crystallize rapidly and independently at each site of the interface with little time to align individual grains. By decreasing the deposition rate, the newly trapped particles could have sufficient time to rearrange into the most energetically favorable hexagonal configuration, effectively minimizing the formation of grain boundaries. Similarly, the competitive interactions (exceeding or matching) between centrifugal force and electrostatic repulsion may drive the epitaxial growth of upper particles on the bottom template^39^, finally leading to the formation of polycrystalline or single-crystalline multilayer, respectively.

In the NaCl-treated suspension, because the strong electrostatic repulsion at short distances could not be easily overcome by the centrifugal force until the upper particles reached 16 layers (Fig. 4d), the crystallization of particles was effectively suppressed at a rapid deposition rate (see Fig. S5), producing quasi-amorphous structure with short-range ordering.

### Binding mechanism

Because the hydration layers on silica surfaces can act as a steric barrier to prevent van der Waals attraction between particles, the assembled structures are not stable and even could be easily destroyed either by liquid turbulence arising from external vibrations or by particle diffusion due to interparticle electrostatic repulsion once the centrifugal force is removed. Moreover, because of the poor spreadability of water over the surface of oil^40^,^41^, it is nearly impossible to transfer the loosely packed particles from the interface to a substrate.

Here, we show that by introducing the third immiscible phase of hydrocarbon mixture, which enables the dehydration of colloidal silica during centrifugation, the assembled structure can be preserved and even reinforced after removal of centrifugal force. It has shown that in the presence of small amount of water, hydrophilic particles suspended in a hydrophobic oil can be bound together by capillary water bridges owing to the combined effects of oil/water interfacial tension and negative Laplace pressure^42^,^43^,^44^. In our case, after dehydration at a high speed centrifugation, the source of water necessary for the formation of water bridges between particles may be originated from a residual water layer, which is adhered to the inner hydration layer due to hydrophobic effect^45^,^46^. A typical value of the capillary attraction force *F*_c_ exerted by a water bridge between two contacting spheres can be estimated by *F*_c_ = 2π*rγ*_ow_cosθ^42^, where *r* = 200 nm is the particle radius, *γ*_ow_ ≈ 13.5 mN/m is the hydrocarbon mixture/water interfacial tension, which is mainly determined by the component with the lowest interfacial tension^47^, θ is the wetting angle and expected to be small on hydrophilic particles (θ ≈ 0). Accordingly, *F*_c_ is calculated to be ~1.7 × 10^−8^ N, which is much larger than the hydration repulsion between two contacting silica particles (estimated as 6 × 10^−10^ N^48^). This indicates that after dehydration, the capillary attraction by a water bridge can pull the particles into more intimate contact by breaking the hydration layers even after the centrifugal force is removed. This liquid/liquid capillary force not only effectively binds the particles together, but enables the pre-shrinkage of colloidal array at the fluid interface, thus making it possible to produce free-standing films that are robust enough to be transferred to a solid substrate and remain crack-free after dying.

**Figure 5:**
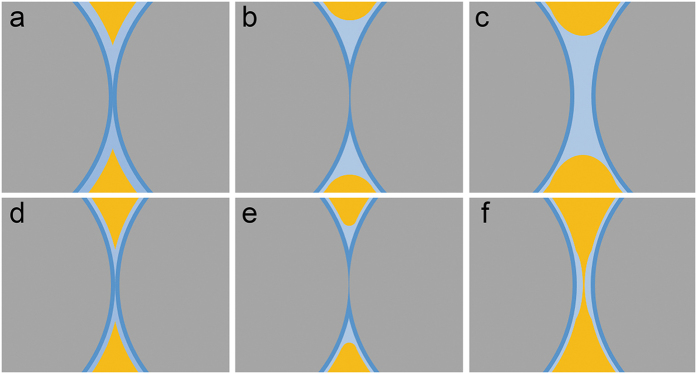
Effect of hydrocarbon mixture/water interfacial tension on the rupture distance of a water bridge between two silica spheres. (**a,b**) In the case of high interfacial tension, a relative thick layer of residual water can be effectively utilized to build a water bridge. (**c**) The water bridge is likely to survive during and after stretching. (**d,e**) In the case of low interfacial tension, a thin layer of residual water can hardly be sucked towards the connecting point. (**f**) The water bridge is readily ruptured upon stretching.

During dehydration process, however, we also need to consider an unfavorable interfacial tension force *F*_t_, which tends to pull the colloidal film together with the separated aqueous phase (see Fig. S6a). The magnitude of this force can be estimated by *F*_t_ ≈2π*Rγ*_ow_, where *R* is the radius of the film and *γ*_ow_ is the interfacial tension between hydrocarbon mixture and aqueous phase at the moment of dehydration. If the hydrocarbon mixture only contains tetrachloroethylene and hexane, both of which have a high interfacial tension against water (44.4 mN/m and 50.4 mN/m at 25 °C, respectively), a large *F*_t_ due to a high *γ*_ow_ value (~48 mN/m) should exceed the downward centrifugal force acting on a thin film and cause curling of the film (see Fig. S6b). Increasing the film thickness by using higher suspension concentration can prevent the curling (see Fig. S6c). Nevertheless, high interfacial tension may also favor a relatively thick layer of residual water due to strong hydrophobic effect (Fig. 5a), wherein the orientational ordering of water molecules at the hydrophobic interface may enhance the bonding strength of water molecules to the particle^49^. Therefore, the energy required for detachment of bound water is increased. The high interfacial tension would also lead to a large negative Laplace pressure (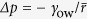), where 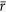 is the radius of curvature of the bridge surface^50^), which could suck the thick residual water layer towards the water bridge (Fig. 5b). Because the rupture distance *δ* of a capillary bridge is proportional to the cube root of the bridge volume *V*, that is *δ* = (1 + θ/2)*V*^1*/*3^ 51, the previously stored water bridge is likely to tolerate large stretching before rupture (Fig. 5c), and the spheres could be pulled back by a strong capillary attraction once the stretching force is removed, being thus responsible for the flexibility of the dehydrated film.

In order to avoid curling and simultaneously to reduce the film thickness, we also attempted to minimize the *F*_t_ by replacing hexane with ethyl acetate, which effectively decreased *γ*_ow_ to as low as that of the interfacial tension of ethyl acetate (6.8 mN/m). However, the film was fragile and easily broken into small fragments after centrifugation (see Fig. S6d). This is presumably because the low interfacial tension would result in a thin layer of residual water, which could not be effectively captured by a water bridge (Fig. 5d,e). Accordingly, the water bridge could be easily ruptured upon stretching (Fig. 5f). These results indicate that a moderate interfacial tension at water/hydrocarbon mixture interface during or after dehydration is necessary, in order to avoid curling and to ensure sufficient strength of the dehydrated film for transferability. This is the reason why butyl acetate, which has a moderate interfacial tension of 13.5 mN/m, was introduced into the mixture of tetrachloroethylene/hexane. When the suspension concentration was above 0.3 wt%, the thickness of colloidal film could be controlled from a dozen microns to several millimeters. However, the single crystalline area tended to decrease as the suspension concentration increased (see Fig. S7).

**Figure 6:**
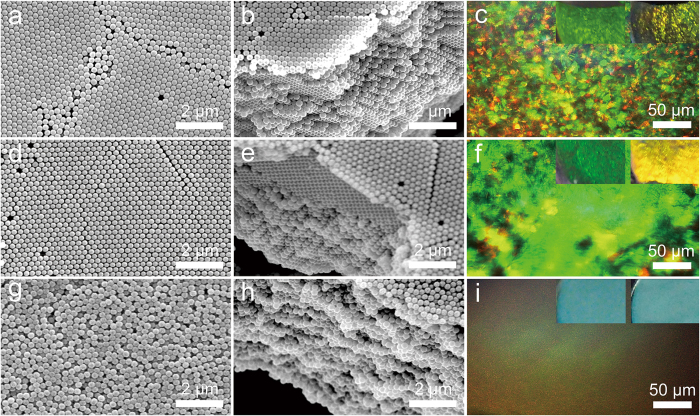
Colloidal films composed of smaller silica spheres. SEM images of the bottom surfaces and the cross-sections, as well as the optical microscope images of the (**a,b,c**) polycrystalline film, (**d,e,f**) single-crystalline film and (**g,h,i**) quasi-amorphous film composed of silica spheres with 270 nm in diameter. Inset: Optical images of the colloidal films taken before drying of the films, at a viewing angle of about 60° (left) and 80° (right), respectively. The concentration of the suspensions was fixed at 0.5 wt%.

Based on the discussion above, when smaller colloidal silica spheres, *e.g.* 270 nm in diameter, were used as the building blocks, the centrifugal force acting on each particle was reduced at the maximum available acceleration of 18,400 g. Thus the assembled colloidal film had a greater tendency to be pulled upwards by *F*_t_ during dehydration. However, the efficiency of utilization of the residual water layer may also be increased due to the higher Laplace pressure owing to a decrease in the curvature radius 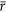. Thus, by using hydrocarbon mixture with lower interfacial tension against water, such as tetrachloroethylene/ethyl acetate mixture, to decrease *F*_t_ during dehydration, we also obtained polycrystalline (Fig. 6a,b,c), single-crystalline (Fig. 6d,e,f) and quasi-amorphous films (Fig. 6g,h,i). These results can be further confirmed by the photographic images of films taken with a camera, which reveal angle-dependent colors for the polycrystalline and single-crystalline films, but angle-independent color for the quasi-amorphous film (see the inserted images in Fig. 7c,f,i).

## Conclusion

We have presented an effective centrifugation-assisted assembly method to create free-standing colloidal films by utilizing the liquid/liquid interfaces of three immiscible phases, including fluorinated oil/water/hydrocarbon solvent mixture. The perfluorinated oil phase provides a low resistance fluid substrate, allowing for the free movement of adsorbed particles towards equilibrium positions. The centrifugal field flattens the perfluorinated oil/water interface, participates in the construction of colloidal multilayer, and also causes the dehydration of the assembled colloidal film by displacing the aqueous phase and hydrocarbon mixture. During assembly process, the hydration layer on silica surface can act as a steric barrier against particle coalescence, while after dehydration, the spontaneous formation of capillary water bridges between particles may lead to a binding and shrinkage toward the center of the dehydrated film. Thus, the assembled films are crack-free and robust enough to be transferred on any planar or curved substrates. Through independent control of the centrifugal force and interparticle electrostatic repulsion, polycrystalline, single-crystalline and quasi-amorphous structures can be readily obtained. This method may also be used for assembly of other colloidal particles into free-standing, crack-free and transferrable films with various structures and potential applications. It may also serve as an easily accessible model for studying the crystallization and phase transformation behaviors of colloidal systems.

## Methods

### Materials

All chemicals used were analytical grade. Hexane, butyl acetate and tetrachloroethylene were mixed in a volume ratio of 3:3:1 at 25 °C, and shaken and sonicated for 10 sec as the hydrocarbon solvent mixture. Perfluorotripropylamine was passed through a nylon syringe filter with a pore size of 0.22 μm to remove possible insoluble impurities. Monodisperse Stöber silica spheres were stored in deionized water before use^52^.

Before preparation, the original silica suspension was centrifuged and washed two times with deionized water, and then dispersed into deionized water at a concentration of 0.3 wt% (the freshly washed suspension). Or, the centrifugation pellet of the original colloidal suspension was dispersed into 5 mM NaCl solution at a concentration of 0.3 wt% (the NaCl-treated suspension).

### Fabrication of colloidal films

Typically, 0.3 mL perfluorinated oil, 1 mL aqueous suspension of colloidal silica and 3 mL hydrocarbon mixture were charged into a 5 mL polypropylene centrifuge tube. The tubes without caps were placed symmetrically in a centrifuge equipped with a fixed angle rotor (H1650-W, Xiangyi Centrifuge Instrument Co., Ltd., China). The centrifugation was carried out at 25 °C in a fume hood. The centrifuge was accelerated to the maximum available acceleration of 18,400 g in 10 or 30 sec, and maintained for 10 min, then decelerated and stopped within 50 sec. After centrifugation, the upper aqueous phase was removed with a Pasteur pipette. The free-standing colloidal films were transferred to a desired substrate from the perfluorinated oil/hydrocarbon mixture interface, and the residual solvents were left to evaporate at ambient pressure and temperature (1 atm, 25 °C) for 3 min. For 270 nm silica spheres, ethyl acetate and tetrachloroethylene were mixed in a volume ratio of 9:1, and the centrifugation was carried out at 18,400 g for 5 min.

### SEM imaging

The free-standing colloidal film was transferred to a double-sided adhesive tape and sputter coated with gold after drying at ambient pressure and temperature. Images of the bottom surfaces and the cross-sections of the colloidal films were obtained on a Philips XL30 SEM operating at an accelerating voltage of 10 kV.

### Optical microscope observation

To observe the internal crystal structure of the colloidal film, the free-standing film was transferred to a thin glass coverslip and covered by a drop of silicone oil to suppress the volatilization of void-filling fluid, then covered with another glass coverslip. The colloidal film was observed under a Hirox KH-7700 digital microscope equipped with a MX-10C co-axial vertical lighting zoom lens, an OL-700II objective lens and an AD-10S Directional Lighting Adapter, at a magnification of ×700.

## Additional Information

**How to cite this article**: Fan, W. *et al.* Centrifugation-assisted Assembly of Colloidal Silica into Crack-Free and Transferrable Films with Tunable Crystalline Structures. *Sci. Rep.* **5**, 12100; doi: 10.1038/srep12100 (2015).

## Supplementary Material

Supplementary Information
