# Supplementary material for: Centrifugation-assisted Assembly of Colloidal Silica into Crack-Free and Transferrable Films with Tunable Crystalline Structures
Source: Sci Rep. 2015 Jul 10;5:12100. doi: 10.1038/srep12100 (PMC4498329; doi:10.1038/srep12100)
Supplement: Supplementary Information [file srep12100-s1.doc]

**Supplementary Information**

Centrifugation-assisted Assembly of Colloidal Silica into Crack-Free and Transferrable Films with Tunable Crystalline Structures

Wen Fan,^1^ Min Chen,^1^ Shu Yang ^2^ & Limin Wu^1*^

^1^Department of Materials Science and State Key Laboratory of Molecular Engineering of Polymers, Advanced Coatings Research Center of Ministry of Education of China, Fudan University, Shanghai 200433, China

^2^Department of Materials Science and Engineering, University of Pennsylvania, 3231 Walnut Street, Philadelphia, PA 19104, USA

*Corresponding author: [lmw@fudan.edu.cn](mailto:lmw@fudan.edu.cn)

Unless otherwise noted, all the following calculations were carried out at 25°C. The following calculations mainly focus on expanding the existing models of interfacial behavior from normal gravity to centrifugation conditions.

1. **Effect of centrifugal acceleration on the background profile of perfluorinated oil/water interface**

First of all, it is necessary to investigate the shape of the perfluorinated oil/water interface during centrifugation, since the background curvature of the interface which originates from the difference in wettability of adjacent liquid on the tube wall, may affect the motion of particles adsorbed at the interface through a well-known meniscus effect.^[1]^ First, the three-phase contact angle $\alpha$ between perfluorinated oil (f), water (w) and the surface of polypropylene centrifuge tube (s) is calculated theoretically, and is related to the interfacial tension ($\gamma$) between each two adjacent phases by Young's equation:

$\cos\alpha=\frac{\gamma_{\text{sw}}-\gamma_{\text{sf}}}{\gamma_{\text{fw}}}$ (S1.1)

Where $\gamma_{\text{fw}}$ is already known, while $\gamma_{\text{sw}}$ and $\gamma_{\text{sf}}$ can be predicted according to the surface energy components method.^[2]^

Briefly, any surface tension $\gamma$, usually against air (a), can be expressed as a sum of components stemming from dispersion forces ($\gamma^{\text{d}}$) and polar forces ($\gamma^{\text{p}}$):

$\gamma=\gamma^{\text{d}}+\gamma^{\text{p}}$ (S1.2)

Besides, the interfacial tension between two phases x and y can be expressed as follows:

$\gamma_{\text{xy}}=\gamma_{\text{xa}}+\gamma_{\text{ya}}-2\sqrt{\gamma_{\text{x}}^{\text{d}}\gamma_{\text{y}}^{\text{d}}}-2\sqrt{\gamma_{\text{x}}^{\text{p}}\gamma_{\text{y}}^{\text{p}}}$ (S1.3)

Therefore, the values of $\gamma_{\text{f}}^{\text{d}}$ and $\gamma_{\text{f}}^{\text{p}}$ are obtained by solving the following equation:

$\gamma_{\text{fa}}=\gamma_{\text{f}}^{\text{d}}+\gamma_{\text{f}}^{\text{p}}$ (S1.4)

$\gamma_{\text{fw}}=\gamma_{\text{fa}}+\gamma_{\text{wa}}-2\sqrt{\gamma_{\text{f}}^{\text{d}}\gamma_{\text{w}}^{\text{d}}}-2\sqrt{\gamma_{\text{f}}^{\text{p}}\gamma_{\text{w}}^{\text{p}}}$ (S1.5)

Where $\gamma_{\text{fw}}$=40.0 mN/m,^[3]^ $\gamma_{\text{fa}}$=15.0 mN/m, $\gamma_{\text{wa}}$=71.9 mN/m, $\gamma_{\text{w}}^{\text{d}}$=21.5 mN/m and $\gamma_{\text{w}}^{\text{p}}$=50.4 mN/m,^[2]^ and the results are $\gamma_{\text{f}}^{\text{d}}$=14.3 mN/m and $\gamma_{\text{f}}^{\text{p}}$=0.7 mN/m.

By knowing $\gamma_{\text{f}}^{\text{d}}$ and $\gamma_{\text{f}}^{\text{p}}$, we can calculate the value of $\gamma_{\text{sf}}$ as follows:

$\gamma_{\text{sf}}=\gamma_{\text{sa}}+\gamma_{\text{fa}}-2\sqrt{\gamma_{\text{s}}^{\text{d}}\gamma_{\text{f}}^{\text{d}}}-2\sqrt{\gamma_{\text{s}}^{\text{p}}\gamma_{\text{f}}^{\text{p}}}$ (S1.6)

Where $\gamma_{\text{sa}}$=30.3 mN/m, $\gamma_{\text{s}}^{\text{d}}$=26.7 mN/m and $\gamma_{\text{s}}^{\text{p}}$=3.6 mN/m,^[4]^ and the result is $\gamma_{\text{sf}}$=3.0 mN/m. Similarly, it can be calculated that $\gamma_{\text{sw}}$=27.3 mN/m.

Finally, according to equation (S1.1), the predicted value of three-phase contact angle $\alpha$ is 52.6°. On the other hand, according to literatures, the background profile $\zeta_{\infty}$ for a circular container is given by:^[5,6]^

$\zeta_{\infty}(r)=\frac{\left[\partial\zeta_{\infty}/\partial r\right]_{\text{ref}}}{qI_{1}(qr_{\mathrm{ref}})}I_{0}(qr)$ (S1.7)

Where the center of the container is located at $r=0$, $r_{\mathrm{ref}}$ is a reference position close to the vertical wall and here it is equal to the inner radius of the centrifuge tube, $I_{0}$ and $I_{1}$ are the zeroth-order and first-order modified Bessel functions of the first kind, respectively, $q=\sqrt{G(\rho_{\text{f}}-\rho_{\text{w}})/\gamma_{\mathrm{fw}}}$ is the inverse capillary length, in which $G=Ng$ is the centrifugal acceleration expressed as multiples ($N$) of gravitational acceleration $g$, $\rho$ represents the liquid density, and $\left[\partial\zeta_{\infty}/\partial r\right]_{\text{ref}}$ is the interface slope at $r=r_{\text{ref}}$. We assume that $\left[\partial\zeta_{\infty}/\partial r\right]_{\text{ref}}=\tan\alpha$, and the increase in centrifugal acceleration will not affect the value of three-phase contact angle $\alpha$.

It is noted that equation (S1.7) is valid only if $\left[\partial\zeta_{\infty}/\partial r\right]_{\text{ref}}^{2}$<<1, in order to satisfy the linear approximation of Young-Laplace equation. Here, we investigate the effect of centrifugal acceleration on the background profile when the three-phase contact angle is small, such as $\left[\partial\zeta_{\infty}/\partial r\right]_{\text{ref}}^{2}=\tan^{2}10^{\circ}\approx0.03$, which satisfies the above condition, and then estimate the effect for a larger contact angle.

**Figure S1** shows the background profiles of perfluorinated oil/water interface at increasing centrifugal acceleration ranging from 1 g to 10,000 g, with $r_{\text{ref}}=0.6\times10^{-2}\text{ m}$, $\rho_{\text{f}}=1,820\text{ kg/m}^{3}$, $\rho_{\text{w}}=1,000\text{ kg/m}^{3}$, $\gamma_{\text{fw}}$ =40.0 mN/m, and $\alpha=10^{\circ}$. To facilitate comparisons, we located the centers of these background profiles at the origin of the coordinate system. It is clear that the centrifugal acceleration has a strong inhibitory effect on capillary rise and capillary length near the wall. When the centrifugal acceleration is greater than 1,000 g, the interface can be considered to be perfectly flat over most of the region, and thus the contribution of the interface curvature can be neglected in this region. Because the interface profile should become flatter when the three-phase contact angle is approaching to 90°, one can speculate that at a large contact angle, such as $\alpha=52.6^{\circ}$ in our case, together with a centrifugal acceleration greater than 1,000 g, we could neglect the background force on the adsorbed particles.


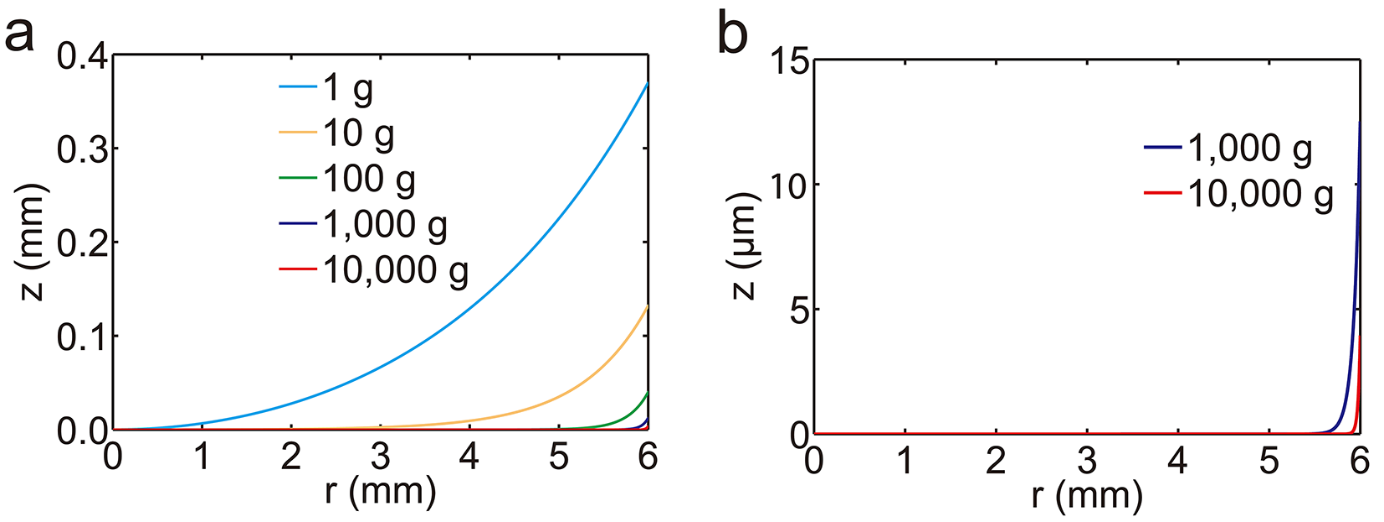


**Supplementary Figure 1.** (a) The background profiles of perfluorinated oil/water interface under increasing centrifugal acceleration ranging from 1 g to 10,000 g. (b) Zoom in of (a) for 1,000 g and 10,000 g.

It is noteworthy that as the perfluorinated oil is capable of preferentially wetting the inner wall of the polypropylene centrifuge tube, there may still be a small capillary rise remaining attached to the wall even under a high centrifugal acceleration. This capillary rise is somewhat necessary because the deposited particles will be collected in a region of low curvature and the concave meniscus can act as the boundary, which confines all the particles within the flat region of the interface. If a convex meniscus is present, such as in the case of using a glass centrifuge tube in which water is capable of preferentially wetting the glass wall, the deposited particles may fall in the gap between the meniscus and the wall, and thus the confining effect is expected to be small. For simplicity, the above analyses neglect the possible effect of non-perpendicular orientation of the interface to the tube wall occurring with the use of a fixed-angle centrifuge rotor.

1. **Calculations of van der Waals attraction force and electrostatic repulsion force between two silica spheres, and the net centrifugal force acting on a setting sphere**

The van der Waals attraction force $F_{\text{vdw}}$ between two spheres *i* and *j* can be expressed as:^[7]^

$F_{\text{vdw}}=-\frac{A_{\text{H}}(4r_{i}r_{j})^{3}}{6}\left\{\frac{d}{\left[d^{2}-\left(r_{i}+r_{j}\right)^{2}\right]^{2}\times\left[d^{2}-\left(r_{i}+r_{j}\right)^{2}+4r_{i}r_{j}\right]^{2}}\right\}$ (S2.1)

Where $r_{i}$ and $r_{j}$ are the radii of sphere *i* and *j* respectively, *d* is the center-to-center distance between two spheres, $A_{\text{H}}$ is the Hamaker constant ($A_{\text{H}}=8.3\times10^{-21}\text{J}$for silica across water^[8]^).

The screened Coulomb repulsion between any two spheres (partially or completely immersed in the aqueous phase) can be estimated as:^[7]^

$F_{\text{ele}}=64\pi\varepsilon_{\text{r}}\varepsilon_{0}\kappa\left(\frac{RT}{zF}\right)^{2}\times\tanh\left(\frac{zF\zeta_{i}}{4RT}\right)\times\tanh\left(\frac{zF\zeta_{j}}{4RT}\right)\times\frac{r_{i}r_{j}}{r_{i}+r_{j}}e^{-\kappa h_{ij}}$ (S2.2)

Where $\varepsilon_{\text{r}}$ is the relative permittivity of water, $\varepsilon_{0}$ is the permittivity of vacuum, $R$ is the gas constant, $T$ is the absolute temperature, $z$ is the valence of the electrolyte, $F$ is the Faraday constant, $\zeta_{i}$ and $\zeta_{j}$ are the zeta potentials of particle *i* and *j* respectively, $h_{ij}$ is the surface-to-surface distance between two spheres, and the Debye-Hückel parameter $\kappa$ can be calculated as follows:

$\kappa=\left(\frac{2000N_{\text{A}}e^{2}I}{\varepsilon_{r}\varepsilon_{0}k_{\text{B}}T}\right)^{1/2}$ (S2.3)

Where $N_{\text{A}}$ is Avogadro's constant, $e$ is the elementary charge, $I$ is the ionic strength of the medium, and $k_{\text{B}}$ is the Boltzmann constant.

The values of $\varepsilon_{\text{r}}$, $\varepsilon_{0}$, $R$, $F$, $N_{\text{A}}$, $e$ and $k_{\text{B}}$ in equation (S2.2) can be obtained from the literature,^[9]^ and the values of the other parameters used in the calculations are listed in **Table S1**.

**Supplementary Table 1.** Some parameters used in the calculation of electrostatic repulsion force.

| **Parameters** | | **Values** |
| --- | --- | --- |
| Particle radius, $r_{i}=r_{j}$ | | 200 nm |
| Ionic valence, $z$ | | 1 |
| Absolute temperature, *T* | | 298.15 K |
| Ionic strength, *I* ^a)^ | 5 mM NaCl suspension | 5×10^-3^ mol/L |
|  | Freshly washed suspension | 1.9×10^-5^ mol/L |
| Zeta potential, $\zeta_{i}=\zeta_{j}$ | 5 mM NaCl suspension | -71 mV |
|  | Freshly washed suspension | -51 mV |

^a)^ The ionic strength of the 5 mM NaCl suspension was estimated as the molar concentration of NaCl. The ionic strength of the freshly washed suspension was estimated from the measured conductivity $K$ (in unit of μs/cm) of the suspension by using a conversion factor of 1.27×10^-6^,^[10]^ that is, $I=1.27\times10^{-6}K$ mol/L. The conductivity and the zeta potential were measured using Malvern Zetasizer Nano ZS90.

Here, we suppose that silica spheres have a critical surface-to-surface approach distance of about 8.8 nm, which is equal to the double thickness of the hydration layer on silica surface. Beyond this distance, the hydration repulsion should disappear and then equation (S2.1) and (S2.2) will be valid.

On the other hand, the net centrifugal force acting on a setting sphere can be calculated as a function of centrifugal acceleration *G*:

$F_{\text{net}}=\frac{4}{3}\text{π}R_{\text{p}}^{3}(\rho_{\text{p}}-\rho_{\text{w}})G$ (S2.4)

The electrostatic repulsion force between two spheres dispersed in 5mM NaCl aq. solution or deionized water is shown in Figure 4b. The van der Waals attraction force between two spheres, and the net centrifugal force acting on a setting sphere are plotted in **Figure S2**.

**
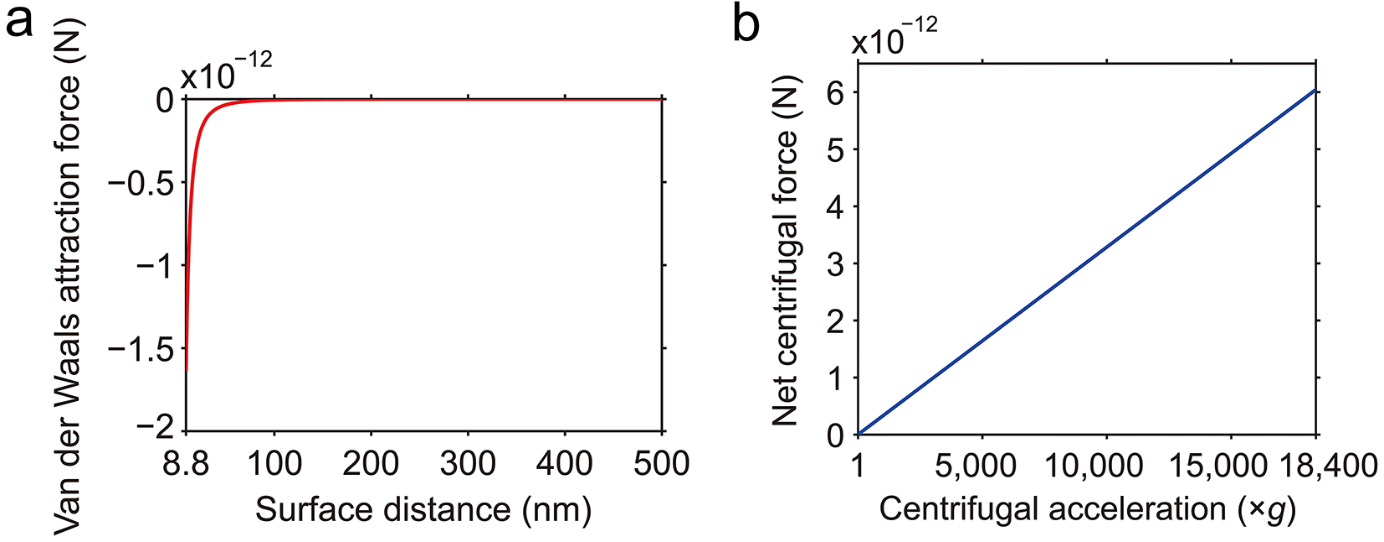
**

**Supplementary Figure 2.** (a) The van der Waals attraction force between two silica spheres, as a function of surface separation distance ranging from 8.8 nm to 500 nm. (b) The net centrifugal force acting on a setting sphere, as a function of centrifugal acceleration.

1. **Calculations of centrifugation-induced capillary attraction force and the energy of capillary attraction between two adsorbed colloidal particles**

In order to calculate the centrifugation-induced capillary attraction force between two adsorbed particles, we need to accurately predict the interfacial deformation created by a single particle. Because the Stöber silica particles are hydrophilic and have a density greater than perfluorinated oil, the perfluorinated oil/water interface must deform to balance the net centrifugal force acting downward on an adsorbed particle. The resulting equilibrium position of a particle at the interface is shown in **Scheme S1**, expressed in a Cartesian coordinate system with the *r*-axis coinciding with the horizontal interface far from the particle and the *z*-axis passing through the center of the sphere.^[5,11]^


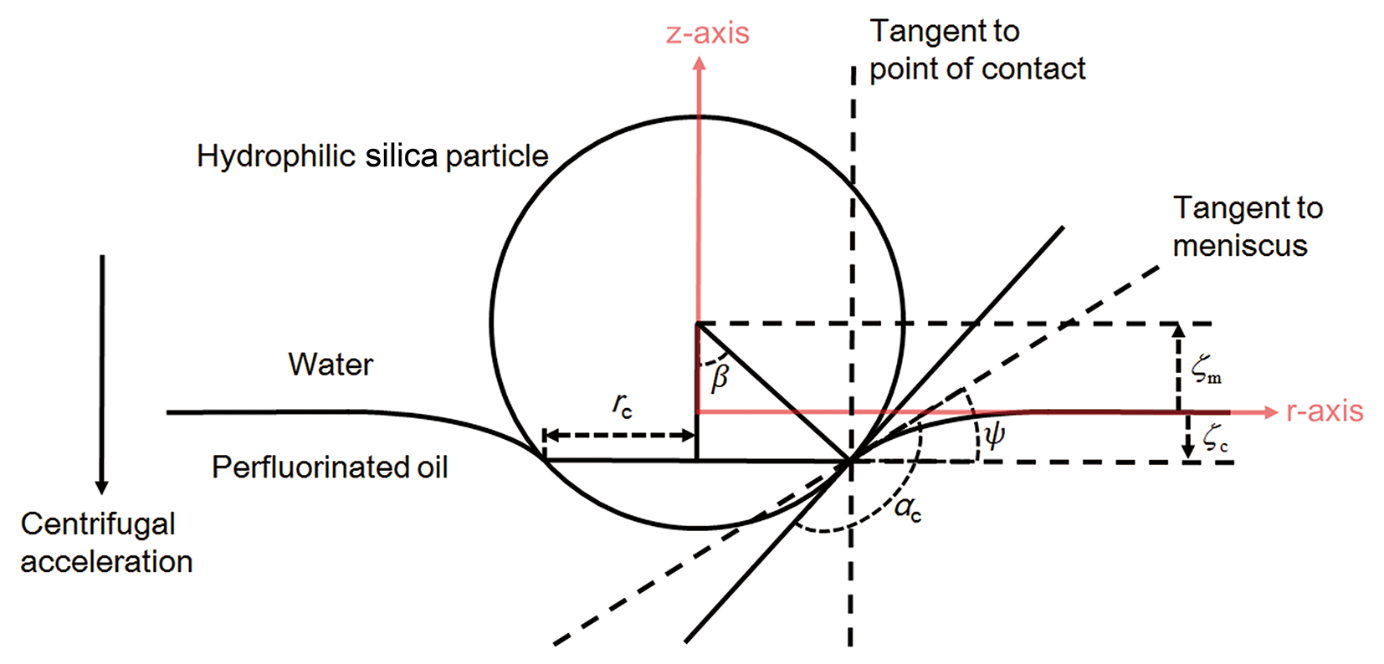


**Supplementary Scheme 1.** Equilibrium position of a silica particle adsorbed at the perfluorinated oil/water interface under a centrifugal field. The particle has a density $\rho_{\text{p}}$ and a radius of $R_{\text{p}}$. $\alpha_{\text{c}}$ is the three-phase contact angle, $\beta$ is the central cone angle, $\psi$ is the slope of the interface at the three-phase contact line, $\psi=\alpha_{\text{c}}+\beta-\pi$. $r_{\text{c}}$ is the radius of the three-phase contact line, $\zeta_{\text{c}}$ is the vertical distance between the horizontal interface and the three-phase contact line, and $\zeta_{\text{m}}$ is the vertical distance between the horizontal interface and the center of the particle.

It is assumed that the silica particle has a smooth and homogeneous surface and the three-phase contact angle $\alpha_{\text{c}}$ is a constant satisfying the Young's equation:

$\cos\alpha_{\text{c}}=\frac{\gamma_{\text{pw}}-\gamma_{\text{pf}}}{\gamma_{\text{fw}}}$ (S3.1)

Where $\gamma_{\text{pw}}$ and $\gamma_{\text{pf}}$ are the interfacial tensions of the particle/water and particle/perfluorinated oil interfaces, respectively. By using the surface energy components method described above and assuming that the Stöber silica particle has the most hydrophilic surface (100% silanol),^[2]^ we can predict the three-phase contact angle $\alpha_{\text{c}}=132.9^{\circ}$.

According to literatures, the equilibrium of vertical forces acting on the particle can be expressed as:^[5,6]^

$\underline{F}_{\text{γ}}+\underline{F}_{\text{G}}+\underline{F}_{\text{b}}=0$ (S3.2)

Where $\underline{F}_{\text{γ}}$ is the vertical component of the interfacial tension force:

$\underline{F}_{\text{γ}}=2\text{π}\gamma_{\text{fw}}R_{\text{p}}\sin\beta\sin\psi\underline{e}_{\text{z}}$ (S3.3)

is the centrifugal force:

$\underline{F}_{\text{G}}=-\frac{4}{3}\text{π}R_{\text{p}}^{3}\rho_{\text{p}}G\underline{e}_{z}$ (S3.4)

$\underline{F}_{\text{b}}$ is the buoyancy force:

(S3.5)

With $P_{u}(z)$ and $P_{l}(z)$ represent the pressure in the upper and lower phase, respectively:

$$P_{u}(z)=P_{0}-G\rho_{\text{w}}z$$

$$P_{l}(z)=P_{0}-G\rho_{\text{f}}z$$

Where $P_{0}$ is the interfacial pressure far from the particle. In addition, for a point on the particle surface, $z$ is expressed as: $z=\zeta_{\text{m}}-R_{\text{p}}\cos\theta$, where $\zeta_{\text{m}}=\zeta_{\text{c}}+R_{\text{p}}\cos\beta$. The final expression for the buoyancy force can be described as follows:

(S3.6)

Thus, equation (S3.2) contains two unknown parameters $\beta$ and $\zeta_{\text{c}}$, which can be determined by considering the interface profile $z(r)$ near the particle and solving the Young-Laplace equation. Briefly, when the Bond number is small (that is $B=(\rho_{\text{f}}-\rho_{\text{w}})GR_{\text{p}}^{2}/\gamma_{\text{fw}}$<<1), the meniscus slope is also small, and thus the linear approximation to the Young-Laplace equation is valid. In our case, when $G\leq20,000\text{ g}$ and $R_{\text{p}}\leq1,000\text{ nm}$, the resulting $B\leq4\times10^{-3}$ is sufficient to satisfy the above condition. Therefore, according to the literatures, the second equation can be given by:^[5,6]^

$K_{0}(qR_{\text{p}}\sin\beta)\sin\psi+q\zeta_{\text{c}}K_{1}(qR_{\text{p}}\sin\beta)=0$ (S3.7)

Where $K_{0}$ and $K_{1}$ are the zeroth-order and first-order modified Bessel functions of the second kind, respectively.

By combining equation (S3.2) and (S3.7), we can calculate the values of $\beta$ and $\zeta_{\text{c}}$ for a given centrifugal acceleration. Then, the centrifugation-induced capillary attraction on the *i*th particle due to the presence of the *j*th particle can be calculated through the linear superposition approximation (LSA) given below:^[5,6]^

$\underline{\text{F}}_{\text{lsa}}^{\text{[}i,j]}=-2\text{π}\gamma_{\text{fw}}Q_{\text{p}}^{2}qK_{1}(qr_{ij})\hat{r}_{ij}$ (S3.8)

Where $r_{ij}$ is the center-to-center distance between two particles, $\hat{r}_{ij}$ is the unit vector along the centerline from particle *j* to particle *i*, and the coefficient $Q_{\text{p}}$ is defined as:

$Q_{\text{p}}=\frac{-\tan\psi}{qK_{1}(qR_{\text{P}}\sin\beta)}$ (S3.9)

Having calculated the centrifugation-induced capillary attraction, the energy of capillary attraction between two particles can be determined as:^[1]^$E(r_{ij})=-\int F(r_{ij})\mathrm{d}r_{ij}$, or:

$E(r_{ij})=-2\text{π}\gamma_{\text{fw}}Q_{\text{p}}^{2}qK_{0}(qr_{ij})$ (S3.10)

**Figure S3** shows the calculation results of centrifugation-induced capillary attraction force and the energy of capillary attraction between two adsorbed particles, respectively. The parameters used for calculations are $\rho_{\text{p}}=2,000\text{ kg/m}^{3}$, $\rho_{\text{f}}=1,820\text{ kg/m}^{3}$, $\rho_{\text{w}}=1,000\text{ kg/m}^{3}$, $\gamma_{\text{fw}}$=40.0 mN/m, $\alpha_{\text{c}}=132.9^{\circ}$ and $R_{\text{p}}$ is chosen as 200, 500 and 1,000 nm for comparison, respectively.


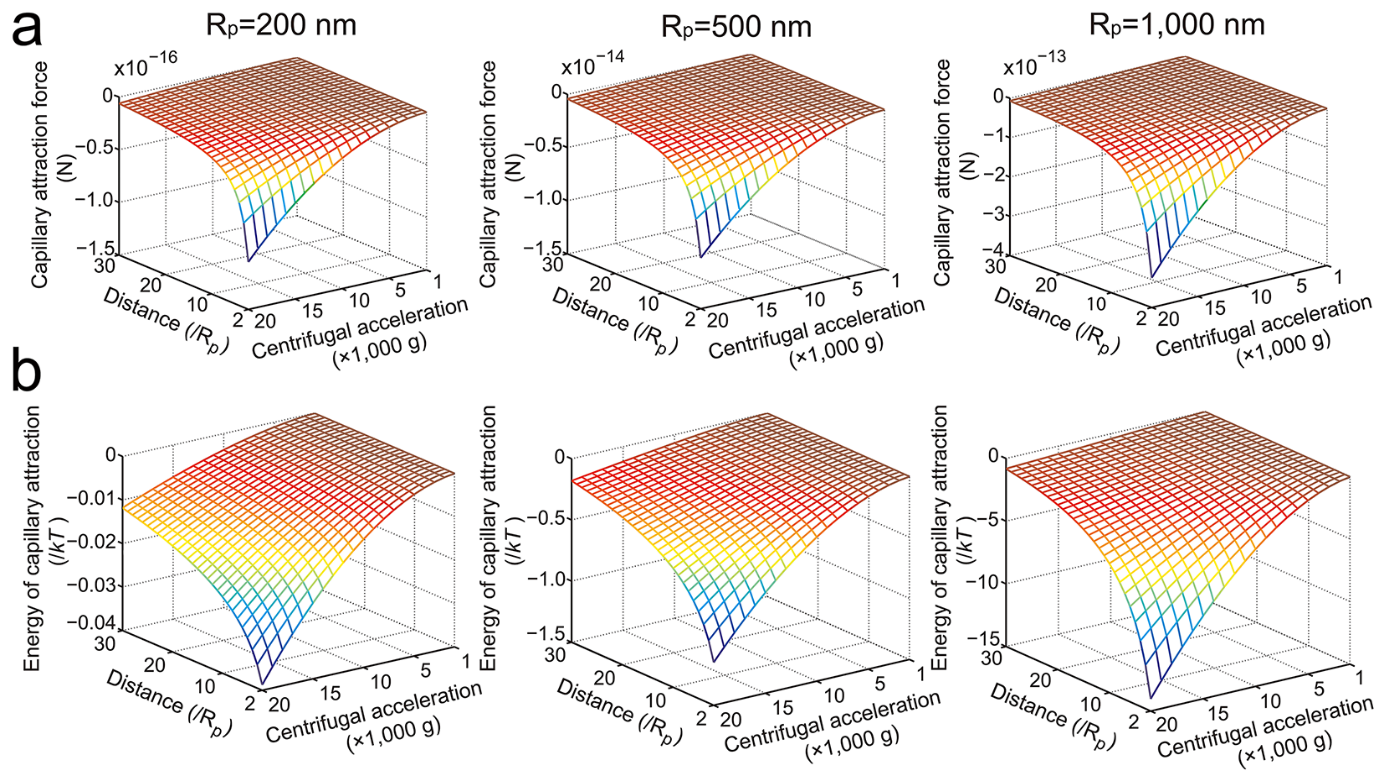


**Supplementary Figure 3.** Calculation results of centrifugation-induced capillary attraction force and the energy of capillary attraction between two adsorbed silica particles. (a) The centrifugation-induced capillary attraction force from silica particles of different radii, and (b) the corresponding energy of capillary attraction (compared to thermal energy $k_{\text{B}}T$) is calculated as functions of centrifugal acceleration (in unit of gravitational acceleration, *g*) and center-to-center distance (in unit of particle radius,$R_{\text{p}}$).

The calculation results indicate that the centrifugation-induced capillary attraction does exist and increases with increasing centrifugal acceleration, especially when the size of silica particles is large (*e.g.* radius ≥ 500 nm). For smaller particles (*e.g.* radius ≤ 200 nm as considered here), the energy of capillary attraction is only a few percent of the thermal energy (*k*_B_*T*) even when the centrifugal acceleration is up to 20,000 g, partly because the deformation of the interface is also inhibited by increasing centrifugal acceleration.

However, the real situation is more complex because the adsorbed particles are subject to an additional *F*_net_ exerted by the upper particles through screened Coulomb repulsion or hydration repulsion, which could further increase the deformation of the interface around the adsorbed particles, and thus increase the centrifugation-induced capillary attraction.

To calculate the effect of additional $F_{\text{net}}$ on the capillary attraction force and the energy of capillary attraction, we can assume that there are *n* layers of upper particles acting on the adsorbed particles and modify the equation (S3.4) as:

$\underline{F}_{\text{G}}=-[\frac{4}{3}\text{π}R_{\text{p}}^{3}\rho_{\text{p}}G+\frac{4}{3}\text{π}R_{\text{p}}^{3}(\rho_{\text{p}}-\rho_{\text{w}})nG]\underline{e}_{z}$ (S3.11)

**Figure S4** shows the capillary attraction force and the energy of capillary attraction between two adsorbed particles (with a radius of 200 nm) under different layers of upper particles.


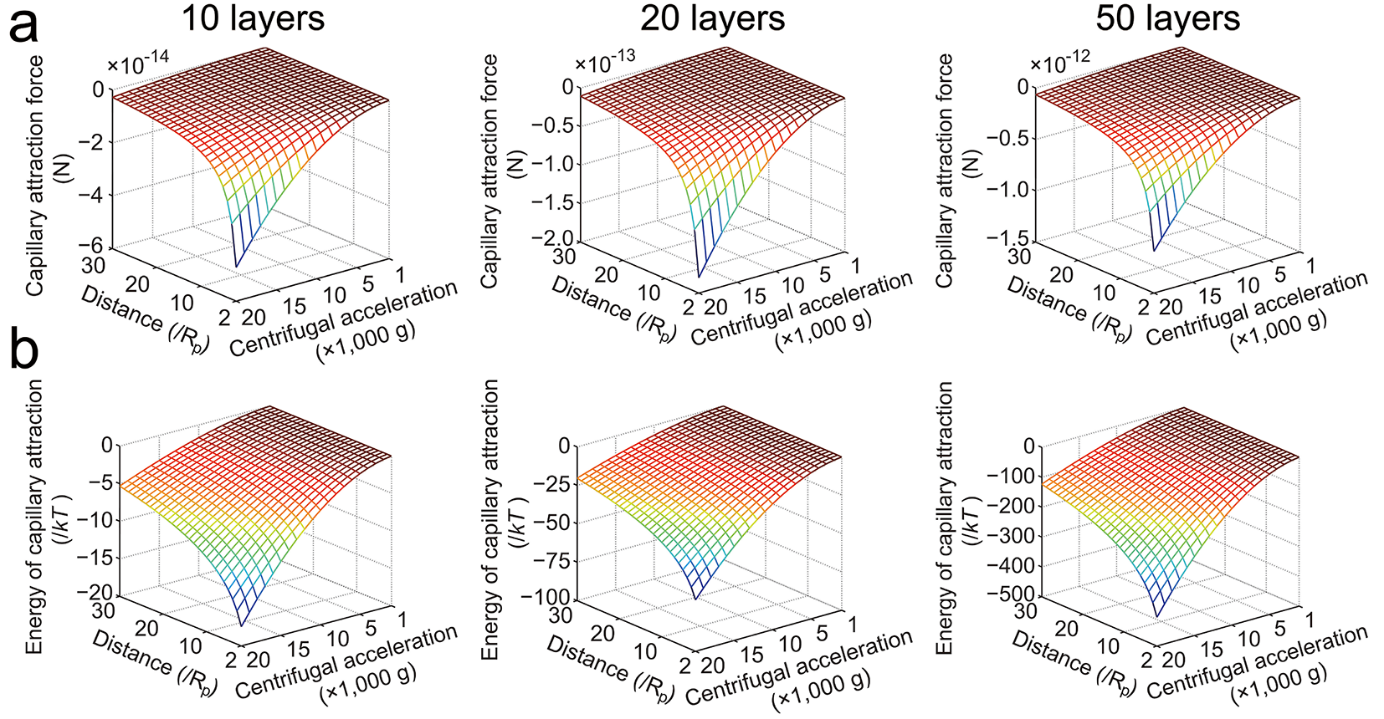


**Supplementary Figure 4.** The effect of additional net centrifugal force on the capillary attraction force and the energy of capillary attraction. (a) The capillary attraction force and (b) the energy of capillary attraction between two adsorbed particles (*R*_p_=200 nm) when they are subject to additional net centrifugal force from 10, 20 and 50 layers of upper particles, respectively.

The calculation results show that the additional *F*_net_ is indeed more effective in increasing the capillary attraction between adsorbed particles, because it directly increases the downward force acted on the adsorbed particles without inhibiting the deformation of the interface. The energy of capillary attraction can increase to the order of tens of *k*_B_*T* when the adsorbed particles are subject to additional 20 layers of upper particles. Nevertheless, the adsorbed particles are densely packed in multilayers, limiting their mobility. Thus, the centrifugation-induced capillary attraction has little influence on the assembly process, especially considering that the possible electrodipping force *F*_el_ could further decrease the capillary attraction.

1. **Effect of acceleration rate of centrifuge rotor on preparation of quasi-amorphous colloidal film**

**
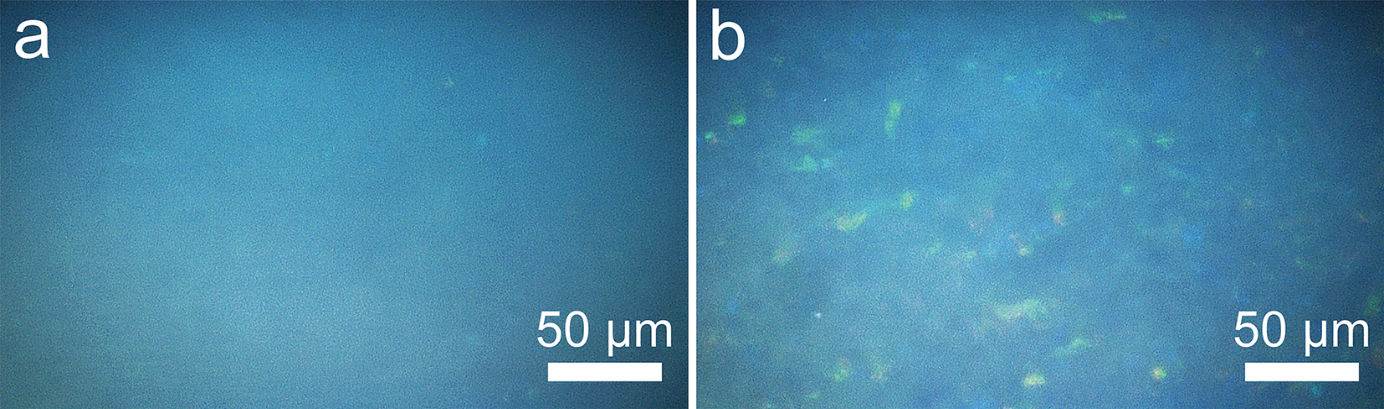
**

**Supplementary Figure 5.** Optical microscope images of colloidal films prepared from 0.3 wt% of 5 mM NaCl aq. suspension with different acceleration time to 18,400 g: (a) 10 seconds and (b) 30 sec.

1. **Effect of hydrocarbon solvent mixture/water interfacial tension on dehydration of colloidal film**


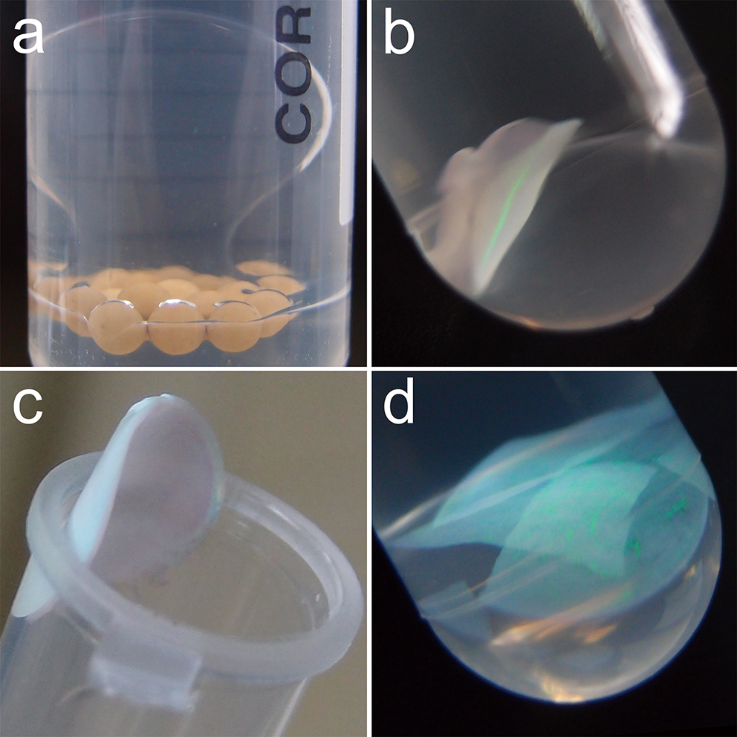


**Supplementary Figure 6. (**a) Photo of dehydration process of hydrophilic particles using molecular sieve under normal gravity condition. **(**b-c) The colloidal films dehydrated from hexane/tetrachloroethylene mixture with a volume ratio of 3:1. **(**b) The film curled when 0.3 wt% of the suspension was used. **(**c) Increasing the concentration of the suspension to 3 wt% avoided the curling arising from dehydration, and the resulting wet film could be bent plastically without breaking. **(**d) The film dehydrated from ethyl acetate/tetrachloroethylene mixture with a volume ratio of 9:1, which was easily broken after centrifugation.

1. **Effect of suspension concentration on preparation of single-crystalline colloidal film**


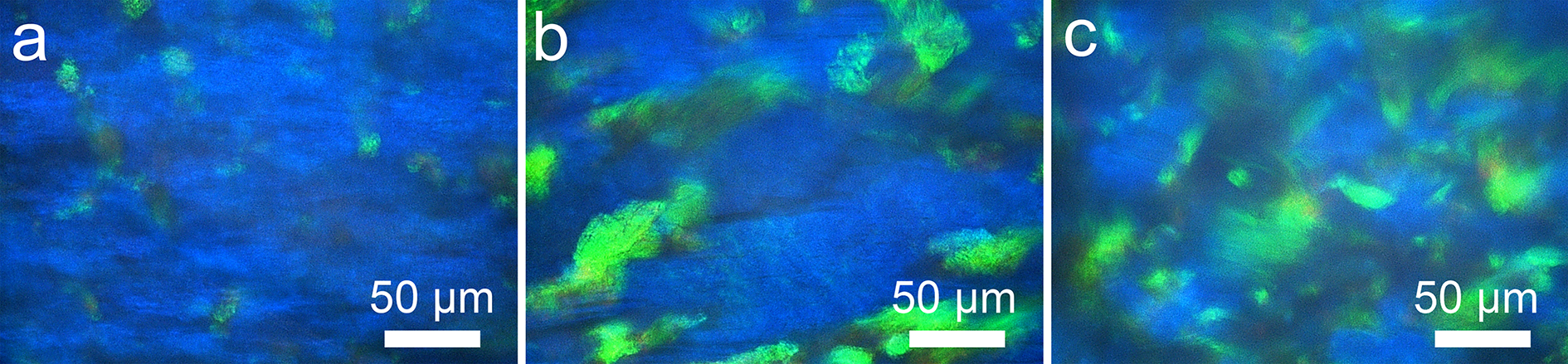


**Supplementary Figure 7.** Optical microscope images of colloidal films assembled from freshly washed suspension with concentrations of (a) 0.3 wt%, (b) 0.5 wt% and (c) 1.0 wt%, respectively. The silica particles have a diameter of 400 nm. The centrifuge was accelerated to the maximum available acceleration of 18,400 g in 30 sec.

The results indicate that the single crystalline area tends to decrease with increasing suspension concentration. This because a higher suspension concentration would indirectly increase the settling rate of silica particles. Further using of centrifuge with lower acceleration rate of centrifuge rotor may allow the preparation of large-area single-crystalline even the suspension concentration is high enough.

**Supplementary References**

1. Vella, D. & Mahadevan, L. The “cheerios effect”. *Am. J. Phys.* 73, 817-825 (2005).
2. Binks, B. P. & Clint, J. H. Solid wettability from surface energy components: relevance to Pickering emulsions. *Langmuir* **18,** 1270-1273 (2002).
3. Boukellal, H., Selimović, Š., Jia, Y., Cristobal, G. & Fraden, S. Simple, robust storage of drops and fluids in a microfluidic device. *Lab Chip*, **9,** 331-338 (2009).
4. Occhiello, E., Morra, M., Morini, G., Garbassi, F. & Johnson, D. On oxygen plasma-treated polypropylene interfaces with air, water, and epoxy resins. II. Epoxy resins. *J. Appl. Polym. Sci.* **42,** 2045-2052 (1991).
5. Vassileva, N. D., van den Ende, D., Mugele, F. & Mellema, J. Capillary forces between spherical particles floating at a liquid-liquid interface. *Langmuir* **21,** 11190-11200 (2005).
6. Vassileva, N. D. *Behavior of two-dimensional aggregates in shear flow* (University of Twente, 2006).
7. Li, J. F., Chen, C. S., Yu, B. Y. & Wei, W. C. J. Simulation of colloidal particle packing for photonic bandgap *J. Am. Ceram. Soc.* **89,** 1257-1265 (2006).
8. Kobayashi, M., Juillerat, F., Galletto, P., Bowen, P. & Borkovec, M. Aggregation and charging of colloidal silica particles: effect of particle size. *Langmuir* **21,** 5761-5769 (2005).
9. Mohr, P. J., Taylor, B. N. & Newell, D. B. CODATA Recommended Values of the Fundamental Physical Constants: 2010a. *J. Phys. Chem. Ref. Data* **41,** 043109 (2012).
10. Zeng, Y. *et al*. Effect of particle size and Debye length on order parameters of colloidal silica suspensions under confinement. *Soft Matter* **7,** 10899-10909 (2011).
11. Singh, P. & Joseph, D. D. Fluid dynamics of floating particles. *J. Fluid Mech.* **530,** 31-80 (2005).
